# “Hadrontherapy for Life” Symposium, Caen, March 10/11, 2025–Strategy for the Future–Pediatric Tumors

**DOI:** 10.1016/j.ijpt.2025.101286

**Published:** 2025-11-26

**Authors:** Jean-Louis Habrand, Semi B. Harrabi, Shigeru Yamada, Zheng Wang, Siamak Haghdoost, Ellie Light, Tatsuya Ohno, Jerôme Doyen, Remi Dendale, Jacques Balosso, Audrey Larnaudie, Anthony Vela, Arnold Pompos, Bradford Hoppe, Anita Mahajan, Juliette Thariat

**Affiliations:** 1 Radiation Oncology Department, François Baclesse Cancer Center, Caen, France; 2 Radiation Oncology Department, Heidelberg University Hospital, Heidelberg, Germany; 3 QST Hospital, National Institutes of Quantum and Radiological Science and Technology, Chiba, Japan; 4 Department of Radiation Oncology, Shanghai Proton and Heavy Ion Center, Shanghai, China; 5 ABTE/ToxEMAC Laboratory, University of Caen Normandy, Caen, France; 6 Radiotherapy Department, The Christie NHS Foundation Trust, Manchester, United Kingdom; 7 Department of Radiation Oncology, Gunma University Graduate School of Medicine, Gunma, Japan; 8 Radiation Oncology Department, Antoine Lacassagne Cancer Center, Nice, France; 9 Radiation Oncology Department, Institut Curie, Paris, France; 10 Radiation Oncology Department, Grenoble University Hospital, Grenoble, France; 11 Radiation Oncology Department, University of Texas Southwestern Medical Center, Dallas, TX, USA; 12 Radiation Oncology Department, Mayo Clinic Florida, Jacksonville, FL, USA; 13 Radiation Oncology Department, Mayo Clinic Rochester, Rochester, MN, USA

**Keywords:** Pediatric tumors, Osteosarcoma, Heavy ion therapy

## Abstract

The symposium “Hadrontherapy for life,” held in Caen, on March 10 and 11, 2025, brought together over 100 international experts of heavy ions particle therapy. Clinical aspects of current indications and future strategies were discussed. If protontherapy remains the cornerstone of current strategies dealing with pediatric malignancies, in order to better spare normal tissues from deleterious effects of radiation, heavier ions such as carbon ions could play a role in selected highly radio-resistant processes such as bone and non rhabdomyosrcomas soft tissue sarcomas. A special mention should be made to helium ions tested since 2021 in Europe that mimic protons with further ballistic selectivity. If immediate and early side effects of heavy ions look modest, long-term tolerance still needs to be carefully evaluated, including risks of carcinogenesis.

## Introduction

The symposium “Hadrontherapy for Life” included discussions by experts on potential clinical indications that would be strategic for future heavy ion radiotherapy (HIRT) programs like those at the CYCLHAD particle therapy center in Caen. Accepted worldwide indications are reported in a separate paper of this issue. For the “strategy session,” we selected two topics for in-depth evaluation in the context of modern strategies and technologies: pancreatic adenocarcinoma and pediatric tumors. The former is exposed separately. The latter look provocative and equally challenging, of note pediatric patients were first treated protons only 3 decades ago with some trepidation, and now the benefits are widely accepted.

## Presentation

Pediatric tumors, that is, children and young adults (AYAs), are rare: less than 5% of all but in constant progression, esp. youngest ones (approx. 0.6% per year).^1^ According to the Global Cancer Oservatory-2022 (including AYAs up to 29 years old) the yearly incidence is approx. 10 800 below 14 years of age and 27 000 above, in US; 16 900 and 43 000, respectively, in Europe, including 1700 and 4700, respectively, in France.^2^ Very polymorphic anatomically, the solid tumors are dominated by brain (25%) and bone/soft tissues sarcomas (11%), predominant above 10 years of age and much more frequent than their adult counterparts (approx.1%, each).^1^, ^2^ Nowadays AYAs are increasingly managed according to pediatric guidelines. Another specificity is their initial presentation, frequently rapidly progressive and more readily metastatic at the onset, which made them particularly dreadful in the past.

## Management

It has considerably improved the outcome since 80s due to the remarkable effectiveness of chemotherapy (CT), especially multi-drug regimen, in this age group. Currently, the overall long-term prognosis is significantly higher (by 20% to 30%)^2^ than that of adults: up to 80%.^1^ However, radiotherapy (RT) remains necessary to the ultimate control in approx. ½ cases, and modern x-rays technologies based on, or inspired from QA monitored beams’ intensity modulation (IMXRT) have substantially improved dose conformation to the target. Nonetheless, tumor radio-resistance and local control remain challenging in part of the indications. As a matter of fact, mortality remains substantial in the CNS and bone/soft tissues that still represent 25% and 16%, respectively, of all reported mortality.^1^ Nonetheless, the major challenge remains nowadays the very long-term toxicity that becomes the major cause of mortality (at 30+ years), along with quality of life and social inclusion.^3^

## Clinical contribution of particle therapy

1. The contribution of proton therapy (PT) has been essential due to a better sparing of healthy tissues compared with x-Rays RT and relative prevention of major sequelae. This is basically related to its original ballistic properties (Bragg curve).
2. Early reports dealing with helium ions (He) have attracted much attention. Ballistically, they exhibit an even sharper lateral penumbra than protons, which should translate in reduced early and late toxicity. First patient has been managed at HIT (Heidelberg), in 2021. In silico studies are actively pursued and still demonstrate a relevant benefit in ependymoma with an integral dose-reduction of 48% and to neuronal structures up to 39% compared with IMXRT,^4^ and in nasopharyngeal angiofibroma.^5^ The reverse side of He is an increased RBE, and untoward “radioactive tail” beyond the Bragg peak (shared with heavier ions although quantitatively less) whose safety deserves confirmation.
3. As far as biological enhanced effectiveness on tumor cells of carbon ion radiotherapy (CIRT), there are no dedicated pediatric CIRT studies, but some (esp. pelvic bone sarcomas) that have lower age-range that allow adolescent patients to receive treatment.^6^ Interesting data have been brought out in Japan and Germany on inoperable or incompletely resected bone sarcomas of the face and pelvis with 85% 5-year survival (OSCAR protocol) following combined CT followed by PT + CIRT.^7^, ^8^ These results compare very favorably with x-rays which historically have been doing very poorly in these radioresistant processes. Noteworthily, full-dose re-irradiation using CIRT concerned also subgroups with attractive findings (Table 1).^7^, ^8^, ^9^, ^10^, ^11^, ^12^, ^13^

**Table 1:** Radiotherapy in trunk/facial bones sarcomas in children and adolescents (for typographic reasons, figures are approximated).

Author (Date) *Journal*	Stage	#Pts *FUp*	RT *(Dose: GyEq)*	CT	Outcome (%)	Toxicity *(RT related)*	Comments
No RT							
Bielack et al (2002) *J Clin Ocol*^9^	Op. & Inop	107 46 m	-	HDMTX based	5YOS: 34 5YEFS: 19 Pelvic: 5YOS: 29% 5YLC: 33%	-	Osteosarcoma
x-rays RT							
Ozaki et al (2003) *J Clin Ocol*^10^	Op. & Inop	*67*	61	HDMX based	5YOS: 27 5YLC: 30 If Inop: 5YOS RT+:29 5YOS RT-: 0 5YLC: 0	NA	OsteosarcomaPelvic bones
PT							
Ciernik et al (2011) *Cancer*^11^	Op & Inop	55	68	HDMTX based & Anthra. based	5YOS: 67 5YLC: 72	Gr3-4:30% (eye, ear, and bowel)	OsteosarcomaTrunk+Facial Low Gr: 63% PT or PT+XR
CIRT, Mix CIRT/PT							
Matsunobu et al (2012) *Cancer*^12^	Inop.	78 42 m	CIRT 53-74 (16 f)	Var.	5YOS: 33 5YLC: 62 If <5 cm: 5YOS:46% 5YLC:88%	Gr3-4: Skin:9% Nerve:5%	Osteosarcoma Trunk (Chest,Spine, and Iliac)
Mohamad et al (2018) *Oncotarget*^13^	Inop	26 *33 m*	CIRT 70 (16 f)	Var.	5YOS: 42 5YLC: 63 >5YOS: 38% If Pelvic: 5YOS: 41% 5YLC: 61%	Gr3-4:15% (skin, nerve, and Bone)	Osteosarcoma Trunk (Chest, Spine, and Iliac)
Schmid et al (2022) *Cancers (Basel)*^7^	Inop & Op:M2 resec Initial & Recurrent (reirradiation)	37 *21 m*	Recurrent: CIRT, 51 (17 f) Initial: PT 45-60 (classic)	Var	2YOS: 86 2YLC: 76 If Initial: 2YOS: 92 2YLC: 83	Gr3-5:0	Ewing Iliac
Seidensaal et al (2022) *Front Oncol*^8^	Inop & Op:M2 resec Initial & Recurrent (reirradiation)	51 *55 m*	CIRT ± PT CIRT ± IMXRT, 60-66 (C:6-8f P,XR:classic)	Var	5YOS: 52 (CIRT+PT:84) If Initial 5YOS: 63 Recurrent 2YOS: 29	Acute Gr3:18 Late Gr3:9	Osteosarcoma Cranio-facial Adults + Children

Abbreviations: Anthra, anthracyclins; EFS, Event-free survival; f, fractions; Gr, grade; Gy Eq, Gy RBE Equivalent; HDMX, high dose methotrexate; Inop, inoperable; LC, local control; m, months; NA, non assessed; Op, operable; OS, overall survival; resec, resected; Y, years; other abbreviations: see text.

## Strategy for the future

1. Exploring in depth ballistical and biological properties of He and initiating dosimetrical and clinical intercomparisons with protons.
2. Screening clinical indications according to their degree of radiosensitivity and initiating pilot studies with HIRT on most radioresistant primaries. These investigations could concern osteosarcomas of trunk and facial bones, inoperable Ewing’s sarcomas, non-RMS soft-part sarcomas, malignant gliomas, rhabdoid tumors, and any other tumor-type (including benign processes) with an unusual poor response to CT.
3. Exploring HIRT-associated toxicity, esp. carcinogenic effects in the long run through clinical and biological investigations.
4. Implementing clinical studies to refine high LET particles indications and modalities of administration. A recent meta-analysis^14^ of CIRT indications, that classified evidence as “strong,” “conflicting,” or “weak,” ranked pediatric tumors in the third group due to the lack of phases III studies. Unfortunately, most families are unwilling to get involved in such evaluations, especially when an innovative type of RT is proposed. Alternative approaches should be considered, supported by the real impetus for collecting data at national and international levels.

## Discussion

1. Undoubtedly low LET particle therapy should remain the cornerstone in the radio therapeutical management of children and adolescents affected with cancer. PT remains nowadays the standard*.* The vast majority of dosimetric intercomparisons between PT and x-rays in children are in favor of the former. This is also increasingly true for clinical data, mostly accumulated in brain and CNS morbid conditions on acute^15^ and late neuroendocrine and cognitive adverse events.^16^, ^17^ Sparing normal tissues from scattered radiation can be achieved much more efficiently with PT than with x-rays, although Hall et al^18^ showed that this was true only with modern proton technologies based on an active scanning delivery (ie, spot scanning). As a matter of fact, most proton and carbon facilities have adopted this technology. Helium ions could become a favorable option in the future. Their wider adoption will depend on further technological advancements leading to compact facilities like existing 1-room proton solutions. HIRT shows promise for cases with either poor prognosis or where conventional local treatment strategies have failed. Preliminary results are promising; however, further long-term evaluation is needed.
2. Although radiation-associated second cancer is a rare event, children are known to be exposed to a much higher risk compared with adults in the long run (10 to 20 years): 3.3 to 11.2-fold with solid second tumors predominating.^19^ Some subgroups are exposed to a higher risk: children affected with hematologic primaries (mainly Hodgkin’s), the youngest ones, and females. Genetic susceptibility also plays a role (eg, ATM, TP53, and NF1 genes). The thyroid and breasts are the most susceptible organs to develop second cancer, unlike bones that are the least susceptible.^19^, ^20^ In large cohorts, the overall risk of secondary cancers has been correlated with the integral dose to the body.^21^ Comparing IMXRT to PT, the ratio of total body dose after cranio-spinal irradiation has been estimated to be 4.7 to 6.3-fold.^22^ Early clinical reports point out the same favorable impression in medulloblastoma and retinoblastoma.^23^, ^24^ While concerns have been raised about the potential for HIRT, particularly CIRT, to increase the risk of secondary malignancies, evidence suggests this risk may not be elevated. Early studies on high LET particles, primarily neutron therapy, suggested a higher risk; however, an inverse relationship between dose per fraction and cancer induction was also observed.^25^ Clinical data, especially in pediatric populations, remain limited, but no increased risk has been reported in the largest European series from Heidelberg or in Japanese cohorts, although follow-up periods are still relatively short. Notably, a large adult cohort study involving 1580 prostate cancer patients demonstrated a significantly lower long-term risk of secondary malignancies following CIRT compared to conventional x-rays RT (*P* ≤ .03).^26^ Furthermore, analyses indicate that secondary neutron production with HIRT is generally lower than with x-ray-based treatments, supporting a potentially favorable safety profile.

## Conclusion

The symposium “Hadrontherapy for Life” clearly showed the clinical interest of high LET particles in addition to, or in combination with, low LET protons to combine ballistic selectivity (to better spare normal tissues from deleterious effects of radiation) and original biological properties (great interest in selected highly radio-resistant processes). A brilliant future looks ahead with innovative projects like multi-ion combinations or flash dose-rate (ie, >100 Gy/sec),^27^ of note innovative equipment will make easier the implementation of heavy ions programs. Pediatric tumors, although a niche quantitatively, have been for long a paradigm for in-depth exploration of radiation toxicity, carcinogenesis, and impact on quality of life. Undoubtedly, this is prone to attract much attention from oncologists, scientists, health authorities, financers, government authorities, future patients, and their families.

## Declaration of Conflicts of Interest

The authors have no conflicts of interest to disclose.
