# Supplementary material for: “Hadrontherapy for Life” Symposium, Caen, March 10/11, 2025–Strategy for the Future–Pediatric Tumors
Source: Int J Part Ther. 2025 Nov 26;18:101286. doi: 10.1016/j.ijpt.2025.101286 (PMC12721058; doi:10.1016/j.ijpt.2025.101286)
Supplement: Supplementary file 1 — Supplementary material [file mmc1.docx]

SUPPLEMENTAL MATERIAL

None
